# Development and validation of a CT-based nomogram for accurate hepatocellular carcinoma detection in high risk patients

**DOI:** 10.3389/fonc.2024.1374373

**Published:** 2024-08-06

**Authors:** Yingying Liang, Hongzhen Wu, Xinhua Wei

**Affiliations:** ^1^ The First Affiliated Hospital of Jinan University, Guangzhou, Guangdong, China; ^2^ Department of Radiology, Guangzhou First People’s Hospital, School of Medicine, South China University of Technology, Guangzhou, Guangdong, China

**Keywords:** hepatocellular carcinoma, diagnosis, nomogram, CT, model

## Abstract

**Purpose:**

To establish and validate a CT-based nomogram for accurately detecting HCC in patients at high risk for the disease.

**Methods:**

A total of 223 patients were divided into training (n=161) and validation (n=62) cohorts between January of 2017 and May of 2022. Logistic analysis was performed, and clinical model and radiological model were developed separately. Finally, a nomogram was established based on clinical and radiological features. All models were evaluated using the area under the curve (AUC). DeLong’s test was used to evaluate the differences among these models.

**Results:**

In the multivariate analysis, gender (p = 0.014), increased Alpha-fetoprotein (AFP) (p = 0.017), non-rim arterial phase hyperenhancement (APHE) (p = 0.011), washout (p = 0.011), and enhancing capsule (p = 0.001) were the independent differential predictors of HCC. A nomogram was formed with well-fitted calibration curves based on these five factors. The area under the curve (AUC) of the nomogram in the training and validation cohorts was 0.961(95%CI: 0.935~0.986) and 0.979 (95% CI: 0.949~1), respectively. The nomogram outperformed the clinical and the radiological models in training and validation cohorts.

**Conclusion:**

The nomogram incorporating clinical and CT features can be a simple and reliable tool for detecting HCC and achieving risk stratification in patients at high risk for HCC.

## Introduction

Hepatocellular carcinoma (HCC) is the fifth most common cancer and the second leading cause of cancer-related mortality worldwide (1, 2). The majority of HCCs occur in patients with hepatitis viruses, alcohol abuse, non-alcoholic fatty liver disease and liver cirrhosis, which are considered as high-risk factors for developing HCC (3, 4). The prognosis of patients with advanced HCC is poor, while the prognosis of patients with early-stage HCC is much better due to effective and curative treatment options, such as resection, percutaneous ablation, or orthotopic liver transplantation (5). Therefore, early detection and accurate diagnosis are important in managing patients with early-stage HCC.

HCC is a unique malignancy that can be diagnosed noninvasively with imaging (6). Once a definitive diagnosis is established, patients may receive priority on the liver transplantation waiting list without mandated pathologic confirmation (7). Although ultrasound (US) is recommended as the main surveillance tool for HCC by most clinical guidelines, its sensitivity for early-stage HCC ranges from 45% to 63%, especially in patients with advanced cirrhosis (8).

Magnetic resonance imaging (MRI) is crucial for the routine diagnosis and evaluation of HCC, however, their wide use may be limited due to the higher cost than that of computed tomography (CT). Thus, CT forms the keystone in the diagnosis of HCC and is recommended as a first-line diagnostic tool for HCC due to its short acquisition time and high spatial resolution. In cirrhotic livers, alterations in hepatic blood flow may cause atypical enhancement patterns (9). Currently, it remains extremely challenging to distinguish HCC from non-HCC lesions because some HCCs show atypical imaging features and some non-HCC lesions may mimic HCC in imaging in patients at high risk of HCC, which may lead to inappropriate treatment (10, 11). In recent years, radiomics, as an emerging methodology in medicine, has shown promising results in detecting HCC in patients at high risk (12, 13). However, the clinical promotion has been limited because it requires special commercial software (14, 15). Thus, there is a need for a simple and precise preoperative approach to detect HCC in patients at high risk.

Our study aimed to identify objective clinical factors and radiological features associated with HCC diagnosis and to develop a new CT-based nomogram that accurately predicts risk in high-HCC risk patients.

### Materials and methods

This retrospective study was approved by the Institutional Review Board with waived requirement for informed consent (Ethical Board Approval Number: “K-2022–004-01”).

**Figure 1:**
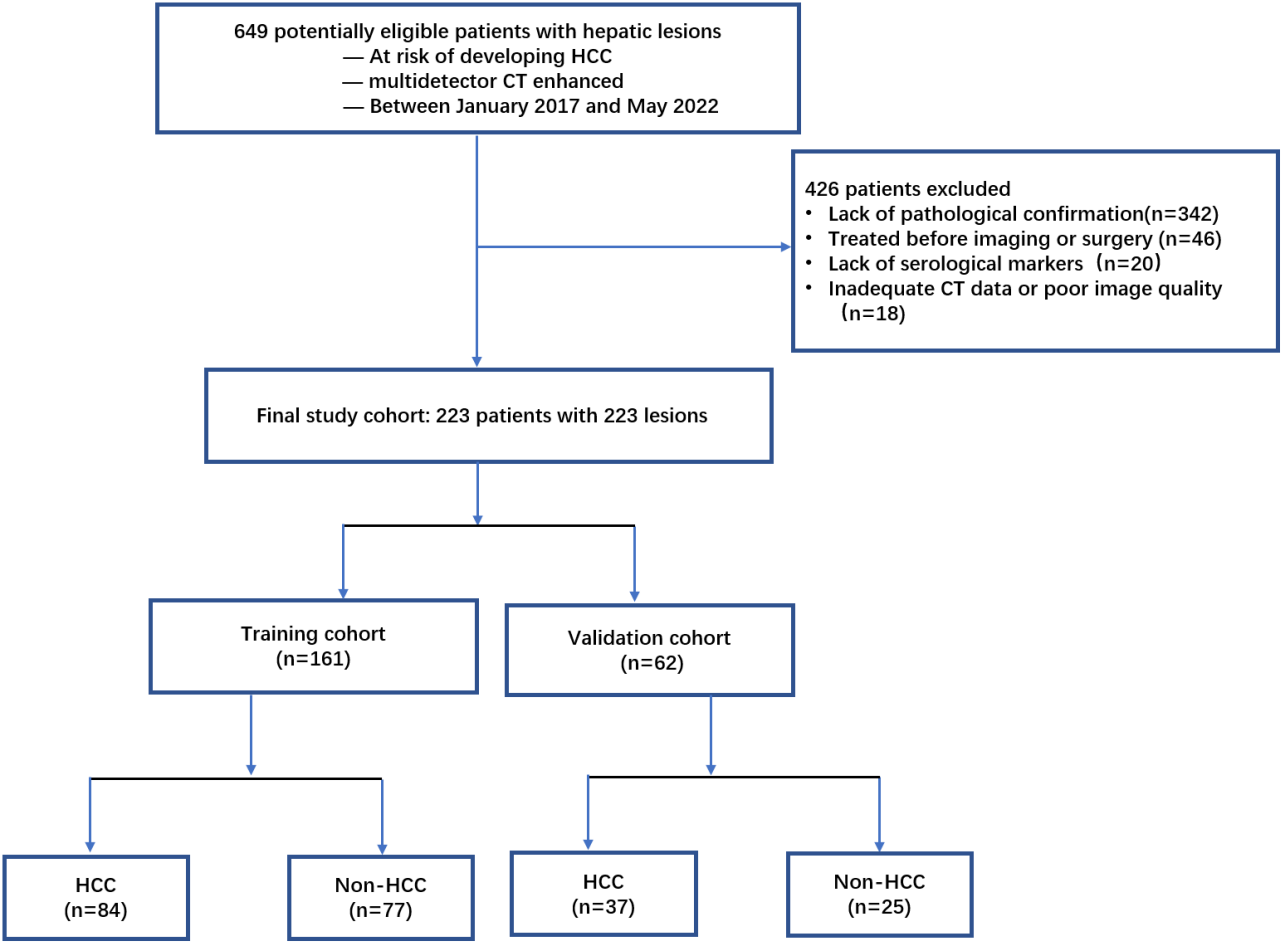
Flowchart of the study population.

A total of 649 patients with liver lesions were enrolled in the study between January of 2017 and May of 2022. Inclusion criteria included: (a) patients who had CT examinations; (b) patients with hepatitis B virus infection, or liver cirrhosis of any cause confirmed histologically or typical radiologically; and (c) those with a pathological diagnosis confirmed within ten days after CT by biopsy or surgical diagnosis. Exclusion criteria included: (a) inadequate confirmation of pathological findings or biopsy (n = 342); (b) treatment given before imaging or surgery (n = 46); (c) inadequate serological markers (n = 20); and (d) inadequate CT data or poor image quality due to movement during the examination (n = 18). Finally, 223 patients were enrolled and randomly assigned to either the training cohort (n = 161) or the validation cohort (n = 62) at a ratio of 7:3 (**Figure 1**).

Baseline demographic data, including age, gender, and laboratory parameters, including Alpha-fetoprotein (AFP), serum total bilirubin, total plasma protein, prothrombin time, and blood platelet levels were collected from each patient’s medical records.

### CT technique

All dynamic acquisition of contrast-enhanced CT exams were performed on a 16-detector CT scanner (Toshiba Aquilion One), 64-detector CT systems (Philips Brilliance), and a 320-detecter CT scanner (Toshiba Aquilion One). The scanning parameters were as follows: 5 mm section thickness, 0.5 s rotation time, 0.9 pitch, 200 mAs tube current, 120 kVP tube voltage, and a 512 × 512 matrix. After an unenhanced CT scan, contrast agent (iodipamide, 370 mg I/mL, Bracco) was injected into the antecubital vein at a rate of 3.5–4.0 mL/s based on their weight (2.0 mL/kg body weight, with a maximal dose of 180ml), followed by 20 mL of saline solution using a power injector. We then obtained the arterial phase (AP, 35–40 seconds), portal venous phase (PVP, 50–60 seconds), and equilibrium phase (EP, 120–250 seconds), respectively.

### CT image analysis

Two abdominal radiologists (who had 6 and 15 years of experience, respectively) retrospectively and independently reviewed the CT images. The readers were blinded to the final pathological diagnoses of the lesions. They assessed the presence of major features of HCC, including non-rim arterial phase hyperenhancement (APHE), non-peripheral washout, and enhancing capsule according to the Liver Imaging Reporting and Data System (LI-RADS) version 2018 (16). They also evaluated the number of lesions, size of the largest lesion, and the presence or absence of necrosis, satellite lesions, internal arteries, and non-enhancing capsules. In addition, tumor with pathological result was evaluated if the liver has different or multiple lesions.

### Statistical analysis

Continuous variables were expressed as the mean ± standard deviation or median, and compared using the Mann–Whitney U test. Categorical variables were expressed as numbers (percentages) and compared using the chi-square test. Clinical and imaging factors, including age, gender, AFP, serum total bilirubin, total plasma protein, prothrombin time, blood platelet levels, tumor size, non-rim arterial phase hyperenhancement (APHE), non-peripheral washout, enhancing and non-enhancing capsule, necrosis, satellite lesions, and internal arteries, were analyzed using the stepwise regression forward method to select the significant independent predictors in the training cohort. Factors whose P values were less than 0.05 in the univariable analysis were inputted into the multivariable logistic regression analysis to identify the independent predictors of HCC. The clinical model (model 1), radiological model (model 2) and clinical-radiologic nomogram (model 3) were constructed by integrating significant clinical factors, significant radiological factors, combined clinical and radiological factors, respectively. The nomogram is based on proportionally converting each regression coefficient in multivariate logistic regression to a 0- to 100-point scale. The points, according to the b coefficient (absolute value) in different variables, are converted to predicted probabilities. The discrimination power of the nomogram was assessed by calibration curves which were assessed with a 1,000 bootstrap resample to measure the accuracy of the nomogram in the training and validation cohorts. A receive operating characteristic (ROC) curve was conducted to evaluate the performance of the different prediction models using area under the curve (AUC). Sensitivity, specificity and accuracy, were then calculated and the DeLong test was used to compare the models’ performances. A decision curve analysis (DCA) was applied to explore clinical usefulness.

The interobserver agreement was analyzed for each feature by using kappa (k) statistics. All statistical analyses were performed using SPSS software (Version 25.0, Chicago, IL, USA) and R software (version 3.6.1). A two-sided p-value of < 0.05 was considered significant in all statistical tests.

## Results

### Demographic data and laboratory parameters

The baseline demographic characteristics of the training and validation cohorts are summarized in **Table 1**. Among the 161 patients in the training cohort, the mean age was 55.28 years, with a range of 20–87 years, and 115 patients (71.4%) were men. Pathologic assessment revealed 84 and 77 cases of HCC and non-HCC lesions, respectively. The malignant non-HCC lesions included cholangiocarcinoma (n = 51), metastasis (n = 2), combined hepatocellular-cholangiocarcinoma (n =1), and epithelioid angiomyolipoma (n =1). The benign non-HCC lesions included focal nodular hyperplasia (n = 12), dysplastic nodules (n =7), and hepatocellular adenoma (n =3). Most lesions were surgically diagnosed, although 16.8% (27/161) lesions were diagnosed after a biopsy. Among the 62 patients in the validation cohort, the mean age was 51.10 years, with a range of 21–82 years, and 44 patients (71.0%) were men. Pathologic assessment revealed 37 and 25 cases of HCC and non-HCC lesions, respectively. The malignant non-HCC lesions included cholangiocarcinoma (n = 13), and metastasis (n = 1). The benign non-HCC lesions included focal nodular hyperplasia (n =8), dysplastic nodules (n =2), and hepatocellular adenoma (n =1). There were 16.1% (10/62) lesions confirmed by biopsy, and the remaining lesions were confirmed by surgery.

**Table 1:** Patient characteristics in the training and validation cohorts.

	Training dataset (n=161)			Validation dataset (n =62)		
	Non-HCC (n=77)	HCC (n=84)	P	Non-HCC (n=25)	HCC (n=37)	P
**Gender(n)**			<0.001*			<0.001*
Male	41(53.2%)	74(88.1%)		11(44.0%)	33(89.2%)	
Female	36(46.8%)	10(11.9)		14(56.0%)	4(10.8%)	
**Age(years)**	55(42,64)	57(50,63.5)	0.509	42(32,60)	57(45,65)	0.045
**Serum total bilirubin(g/L)**	15.7(11.1,25)	34.5(19.25,67.1)	<0.001*	16.1(11.8,72.2)	24(17.7,35)	0.134
**Total plasma protein(g/L)**	67.1(60.6,73.4)	52.9(17.7,65)	<0.001*	72.8(40.2,77.7)	64.4(43,71.7)	0.166
**Prothrombin time(s)**	13.6(13,14.2)	13.6(12.7,14.6)	0.755	13.2(12.8,14)	14(12.8,14.8)	0.175
**Blood platelet(10g/L)**	245(196,290)	171(135.5,229.5)	<0.001*	258(205,341)	172(117,210)	<0.001*
**AFP(ng/ml)**			<0.001*			<0.001*
Negative	62(80.5%)	18(21.4%)		21(84.0%)	7(18.9%)	
Positive	15(19.5%)	66(78.6%)		4(16.0%)	30(81.1%)	
**Maximum dimension (cm)**	5.6(3.4,8.4)	5.15(3.5,7.7)	0.907	6.8(4.7,10.8)	6.8(4.5,8.7)	0.434

AFP, Alpha-fetoprotein; HCC, hepatocellular carcinoma. * P<0.05, significant difference between both groups.

### Interobserver agreement for CT features

The CT features of all patients are shown in **Table 2**. Patients with HCC were more likely to have non-rim APHE (p < 0.001), washout (p < 0.001), enhancing capsules (p < 0.001), necrosis (p < 0.001), and internal arteries (p < 0.001) in the training cohort.

**Table 2:** CT features in the training and validation cohorts.

	Training dataset (n=161)			Validation dataset (n =62)		
	Non-HCC (n=77)	HCC (n=84)	P	Non-HCC (n=25)	HCC (n=37)	P
**Non-rim APHE**			<0.001*			0.007*
Negative	43(55.8%)	7(8.3)		11(44.0%)	5(13.5%)	
Positive	34(44.2%)	77(91.7%)		14(56.0%)	32(86.5%)	
**Washout**			<0.001*			0.001*
Negative	67(87.0%)	18(21.4%)		19(76.0%)	12(32.4%)	
Positive	10(13.0%)	66(78.6%)		6(24.0%)	25(67.6%)	
**Enhancing capsule**			<0.001*			<0.001*
Negative	69(89.6%)	29(34.5%)		25(100.0%)	7(18.9%)	
Positive	8(10.4%)	55(65.5%)		0(0.00)	30(81.1%)	
**Necrosis**			<0.001*			<0.001*
Negative	27(35.1%)	5(6.0%)		13(52.0%)	2(5.4%)	
Positive	50(64.9%)	79(94.0%)		12(48.0%)	35(94.6%)	
**Satellite lesions**			0.431			0.351
Negative	54(70.1%)	54(64.3%)		19(76.0%)	24(64.9%)	
Positive	23(29.9%)	30(35.7%)		6(24.0%)	13(35.1%)	
**Internal artery**			<0.001*			0.162
Negative	37(48.1%)	7(8.3%)		7(28.0%)	4(10.8%)	
Positive	40(51.9%)	77(91.7%)		18(72.0%)	33(89.2%)	
**Nonenhancing “capsule”**			0.426			1.000
Negative	71(92.2%)	80(95.2%)		24(96.0%)	36(97.3%)	
Positive	6(7.8%)	4(4.8%)		1(4.0%)	1(2.7%)	

HCC, hepatocellular carcinoma; APHE, arterial phase hyperenhancement. *P<0.05, significant difference between both groups.

The interobserver agreement for CT features was fair for the maximum dimension (k = 0.43), substantial for the nonenhancing capsule (k = 0.75) and almost perfect for non-rim APHE (k = 0.89), washout (k = 0.90), necrosis (k = 0.90), internal arteries (k = 0.90), enhancing capsules (k = 0.95), and satellite lesions (k = 0.95).

### Development of the prediction model

**Figure 2:**
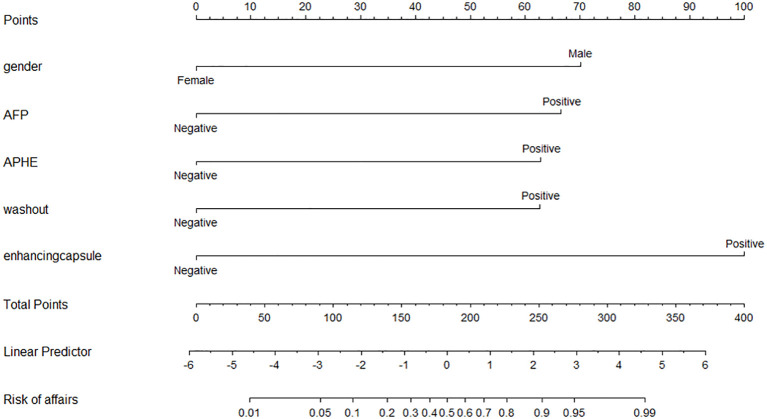
Nomogram for estimating the probabilities of HCC.

Multiple logistic regression analysis revealed that gender (OR = 0.095, 95% CI = 0.015–0.615, p = 0.014), increased AFP (OR = 5.683, 95% CI = 1.358–23.778, p = 0.017), non-rim APHE (OR = 9.619, 95% CI = 1.683–54.971, p = 0.011), washout (OR = 8.231, 95% CI = 1.614–41.978, p = 0.011), and enhancing capsules (OR = 19.136, 95% CI = 3.478–105.291, p = 0.001) were HCC predictors (**Table 3**). A nomogram was constructed from these variables to construct a quantitative and predictive tool (**Figure 2**). Among these significant features, enhancing capsule showed the highest OR and was the best predictor of HCC according to the nomogram.

**Table 3:** Multivariable analysis with logistic regression in the training cohort including clinical and radiological variables.

	B	P value	OR	95% CI	
				Lower	Upper
**Serum total bilirubin**	0.015	0.185	1.016	0.993	1.039
**Total plasma protein**	-0.012	0.439	0.988	0.959	1.018
**Blood platelet**	-0.009	0.065	0.991	0.981	1.001
**Gender**	-2.357	0.014*	0.095	0.015	0.615
**AFP**	1.737	0.017*	5.683	1.358	23.778
**Non-rim APHE**	2.264	0.011*	9.619	1.683	54.971
**Washout**	2.108	0.011*	8.231	1.614	41.978
**Enhancing capsule**	2.952	0.001*	19.136	3.478	105.291
**Necrosis**	2.052	0.051	7.784	0.991	61.176
**Internal artery**	1.569	0.096	4.801	0.756	30.485

CI, confidence interval; OR, odds ratio.

### Predictive performance and validation of the prediction model

**Figure 3:**
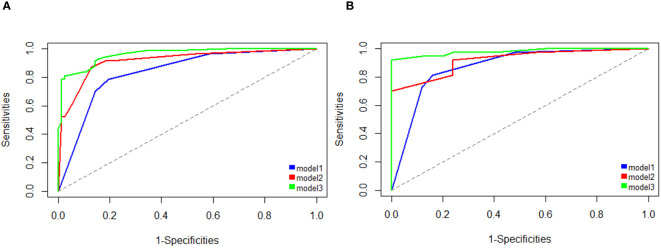
Area under the receiver operating characteristic curve (AUC) analysis shows better performance for detecting HCC using the clinical-radiologic nomogram (model 3) compared with the clinical model (model 1) and radiologic model (model 2) on training cohort **(A)** and testing cohorts **(B)**.

The independent risk factors (including gender and increased AFP) for HCC were used to construct the clinical model. The clinical model predicted HCC with an AUC of 0.838 (95% CI, 0.778–0.897) in the training cohort, and 0.873 (95% CI, 0.782–0.964) in the validation cohort (**Figure 3**). The sensitivity, specificity, and accuracy of the model for the training cohort were 80.5%, 78.6%, and 0.795, respectively, whereas those of the validation cohort were 84.0%, 81.1%, and 0.823, respectively (**Table 4**).

**Table 4:** The specific predictive performances of models for HCC.

Model	Cohort	Criterion			
		AUC (95% CI)	Sensitivity	Specificity	Accuracy
**Clinical**	Training	0.838 (0.778~0.897)	0.805 (0.717~0.894)	0.786 (0.698~0.873)	0.795 (0.793~0.797)
	Testing	0.873 (0.782~0.964)	0.840 (0.696~0.984)	0.811 (0.685~0.937)	0.823 (0.818~0.827)
**Radiological**	Training	0.917 (0.874~0.961)	0.870 (0.795~0.945)	0.869 (0.797~0.941)	0.870 (0.868~0.871)
	Testing	0.918 (0.854~0.983)	0.760 (0.593~0.927)	0.811 (0.685~0.937)	0.790 (0.785~0.796)
**Clinical-radiologic nomogram**	Training	0.961 (0.935~0.986)	0.974 (0.938~1)	0.810 (0.726~0.893)	0.888 (0.887~0.889)
	Testing	0.979 (0.949~1)	1.000 (1~1)	0.919 (0.831~1)	0.952 (0.95~0.953)

Three radiological characteristics, including non-rim APHE, washout, and enhancing capsules, were used to construct the radiological model. The model yielded AUC of 0.917 (95% CI, 0.874–0.961) and 0.918 (95% CI, 0.854–0.983) in the training and validation cohort, respectively (**Figure 3**). The sensitivity, specificity, and accuracy of the model for the training cohort were 87.0%, 86.9%, and 0.870, respectively, whereas those of the validation cohort were 76.0%, 81.1%, and 0.790, respectively (**Table 4**).

The clinical-radiologic nomogram was constructed which combined the two clinical and three radiological characteristics with different score based on their β coefficient, yielded the AUC values of 0.961 (95% CI: 0.935–0.986) with a sensitivity of 97.4%, a specificity of 81.0%, and 0.979 (95% CI: 0.949–1) with a sensitivity of 100.0%, a specificity of 91.9%, in the training and validation cohorts (**Figure 3**). The clinical-radiologic nomogram showed the highest AUC than in the clinical and radiological model in the training cohort (p = 0.041 [clinical vs. radiological model], 0.001[clinical vs. clinical-radiologic nomogram], 0.005 [radiological vs. clinical-radiologic nomogram], respectively), and validation cohort (p = 0.421 [clinical vs. radiological model], 0.010 [clinical vs. clinical-radiologic nomogram], 0.012 [radiological model vs. clinical-radiologic nomogram], respectively).

**Figure 4:**
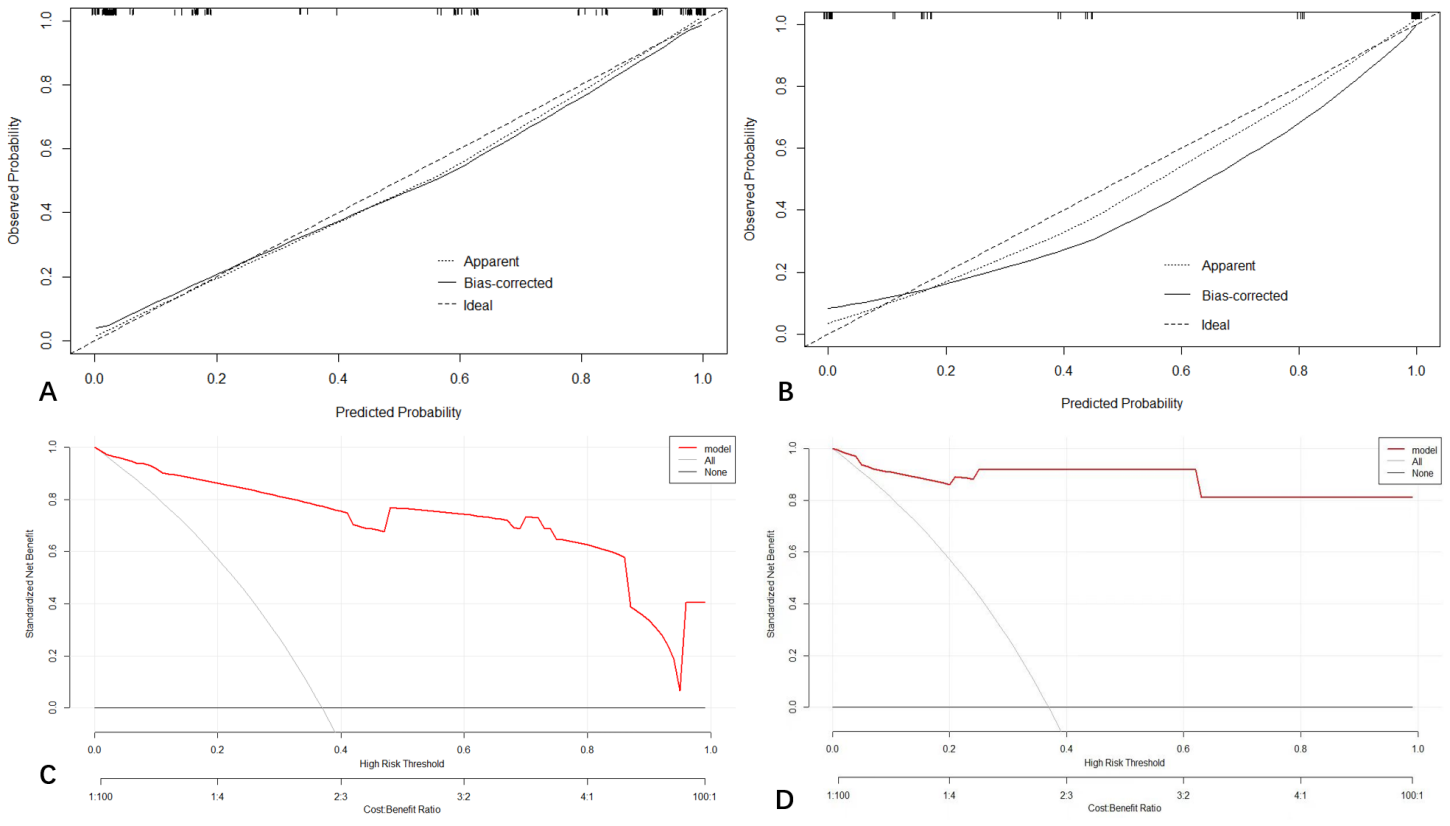
Calibration curve demonstrating how predictions from the model to the actual observed probability on training cohort **(A)** and testing cohort **(B)**. Decision curve analysis (DCA) for the nomogram on training **(C)** and validation cohort **(D)**.

The calibration curve of the prediction model is shown in **Figure 4** indicating that the nomogram is in good agreement with the observations of HCC diagnosis in patients at high risk for HCC.

### Decision curve analysis

The decision curve analysis (DCA) for the nomogram revealed that our prediction nomogram was better able to predict HCC potential than either the treatment or no treatment schemes with the threshold probability >0.1 in the training cohort and in the range between 0 to 1.0 in the validation cohort (**Figure 4**).

## Discussion

In the present study, it showed that gender, elevated AFP level, positive non-rim APHE, washout, and enhancing capsule were independent, significant parameters predicting HCC. Based on these significant clinical factors and radiological features, we constructed and validated a noninvasive nomogram to accurately identify HCC from other hepatic lesions in patients at high risk of HCC. This nomogram showed good predictive ability of HCC in both training (AUC = 0.961) and validation cohorts (AUC = 0.979), which outperformed the clinical and radiological model with a good calibration and clinical applicability. This nomogram was easy to use and facilitated the preoperative detection of HCC for clinicians in order to avoid overdiagnosis and overtreatment.

Our study indicated that enhancing capsule was the best single predictor for diagnosing HCC according to the nomogram, consistent with previous reports (17, 18). Enhancing capsule reportedly has the highest specificity (88%–96%) for diagnosing HCC (17–19). Although enhancing capsule is not evaluated according to the American Association for the Study of Liver Diseases (AASLD) (20) and European Association for the Study of the Liver (EASL) (21) guidelines, it is recognized as a major feature in the LI-RADS algorithm, which could increase the sensitivity (18, 22) and specificity (23) of diagnosing HCC.

Among the three CT features, non-rim APHE had the highest sensitivity (91.7%, 77/84) for diagnosing HCC, which reflects the neoangiogenesis and accelerates the carcinogenesis (24). Although non-rim APHE is considered a crucial imaging feature for diagnosing HCC (24), it is non-specific because it could also be detected in other malignant or benign lesions (25). Recent research has reported that non-rim APHE had the highest sensitivity (85%–94%) (17, 18) but lower specificity (58%–64%) (17, 26) for diagnosing HCC, which was consistent with our results. Meanwhile, Granata et al. confirmed that non-rim APHE could be found in most of the dysplastic nodules (70% [17/24]) in their study population (25). Therefore, attention should be taken in using this feature alone which should lead to false HCC diagnosis.

Washout is the third independent CT feature for diagnosing HCC. There is a discrepancy in washout performance. De Gaetano et al. (17) reported that washout had a high sensitivity (88.2%), but a low specificity (42.3%) for diagnosing HCCs. However, Sangiovanni et al. (26) reported that washout was an effective means of distinguishing HCC from other liver lesions, with a high specificity (100.0%) but a low sensitivity (53.0%). Interestingly, we found that washout was an important independent risk factor of HCC, with a high specificity (87.0%) but a moderate sensitivity (78.6%). Many recent studies have demonstrated that washout combined with non-rim APHE could increase specificity and positive predictability for early HCC diagnosis (18).

AFP is the most widely used tumor marker for diagnosis and evaluation of HCC in clinical practice (27). However, the AASLD does not recommend AFP for the early detection of HCC (20). Previous research has reported that elevated AFP occurred in only 40–65% of HCC patients, while others had normal AFP levels, particularly during the early stages of the disease (28). In addition, many other studies found that an elevated AFP level also occurred in other malignancies or benign liver lesions (29). In our study, gender also had a detrimental effect on HCC diagnosis. Males were more prone to hepatocarcinogenesis, with a prognosis that is worse than in females.

Currently, various imaging models for HCC detection have been described in the literature, especially radiomics models. A study involving 102 patients with liver tumors defined as LR-M based on LI-RADS developed a MRI-based radiomics model to classify HCC and non-HCC tumors with AUC of 0.884 and 0.873, respectively in the training and validation sets (10). Xu et al. found that the deep learning model based on multiphase CT has the potential in accurately classifying HCC from non-HCC from high-risk liver lesions (LI-4/5/M) with AUC of 0.887 and 0.808 (30). Another previous study developed a deep convolutional neural network-ultrasound (DCNN-US) model to classify HCC from focal hepatic lesions, which exhibited high sensitivity and specificity and outperformed radiologists’ visual assessments (31). Undoubtedly, radiomics is important for the diagnosis of HCC with satisfactory model performance. However, it is time-consuming and requires large sample sizes to validate their generalizability. Therefore, to develop a simple and practical model is urgent for radiologists to detect HCC. Our study demonstrated significantly higher performance of the clinical-radiologic nomogram than the clinical or radiological model in detecting HCC without the use of complex software and postprocessing techniques. The AUCs of the training and validation sets were 0.961 and 0.979, respectively, indicating that the nomogram showed good discrimination capability which may aid in the risk stratification and treatment of HCC patients.

There are some limitations to our study. First, it was a retrospective study with a small sample size, especially benign lesions including limited number of regenerative/dysplastic nodules, which may cause potential selection bias. Our work must be validated via prospective studies with larger sample sizes. Second, this was a single-center study, and multi-center validation is required to confirm the nomogram’s reproducibility. Third, a selection bias may affect the validity of the study because biopsy may exclude the possibility of combined hepatocellular-cholangiocarcinomas due to sampling error only the cholangiocarcinoma portion was sampled. Fourth, noisy labels exist widely in CT images which may be contributed to statistical noise, structure noise, artifact noise, and various scanned parameters. The suspected HCC was detected and characterized relying on contrast between liver lesion and background seen in different phases of CT (32–34). Some tumors with variable vascular dynamics may be challenging to detect regardless of the phase due to the different noise levels. Thus, it would be of interest to assess the performance of our nomogram using different noisy labels in real practice. Finally, including only patients with a pathologic diagnosis is a design flaw because most HCC patients end up being diagnosed without recourse to a pathological diagnosis. This is very well illustrated by the larger size of the included tumors.

In conclusion, our study presented a nomogram based on gender, increased AFP, positive non-rim APHE, washout, and enhancing capsule to easily and effectively detect HCC at high risk for this disease, allowing clinicians to rapidly evaluate the risk of HCC and reduce unnecessary surgery. Future studies are needed to externally validate the current model.

## Data availability statement

The raw data supporting the conclusions of this article will be made available by the authors, without undue reservation.

## Ethics statement

The studies involving human participants were reviewed and approved by Ethical Board Approval Number: K-2022-004-01cs committee.

## Author contributions

YL: Conceptualization, Data curation, Funding acquisition, Methodology, Software, Writing – original draft. HW: Conceptualization, Data curation, Formal analysis, Funding acquisition, Software, Validation, Writing – review & editing. XW: Conceptualization, Funding acquisition, Supervision, Writing – review & editing.
